# Predict and prevent microvascular complications of type 2 diabetes: a cross-sectional and longitudinal study in Chinese communities

**DOI:** 10.3389/fendo.2025.1541663

**Published:** 2025-03-31

**Authors:** Zhaoxiang Liu, Lianhao Zhou, Wenhui Zhao, Lixia Jin, Jinping Zhang, Yajing Zhang, Yufeng Li, Guixia Deng, Jiquan He, Xinghua Zhao, Wenli Zheng, Yong Tian, Ji Wu, Jianzhong Xiao, Jiandong Gao

**Affiliations:** ^1^ Department of Endocrinology, Beijing Tsinghua Changgung Hospital, School of Clinical Medicine, Tsinghua Medicine, Tsinghua University, Beijing, China; ^2^ Department of Electronic Engineering, Tsinghua University, Beijing, China; ^3^ Department of Endocrinology, China-Japan Friendship Hospital, Beijing, China; ^4^ Department of Endocrinology, Beijing Pinggu District Hospital, Beijing, China; ^5^ Department of Endocrinology, Beijing Pinggu District Yukou Community Central Health Center, Beijing, China; ^6^ Department of Endocrinology, Beijing Pinggu District Xiagezhuang Township Hospital, Beijing, China; ^7^ Department of Endocrinology, Beijing Huairou District Yangsong Township Hospital, Beijing, China; ^8^ Department of Endocrinology, Beijing Daxing District Yinghai Community Central Health Center, Beijing, China; ^9^ Department of Endocrinology, Beijing Huairou Hospital, Beijing, China; ^10^ Center for Big Data and Clinical Research, Institute for Precision Medicine, Tsinghua University, Beijing, China; ^11^ Center for Intelligent Healthcare, Institute for Precision Medicine, Tsinghua University, Beijing, China

**Keywords:** type 2 diabetes, machine learning, diabetes kidney disease, diabetic retinopathy, diabetic peripheral neuropathy

## Abstract

**Purpose:**

This study investigates the incidence, predictors, and preventive strategies for microvascular complications in type 2 diabetes patients in community settings.

**Methods:**

Data were collected from 3,008 type 2 diabetes patients enrolled across 31 clinics in Beijing and Hebei. Prevalence and incidence of diabetic kidney disease (DKD), diabetic retinopathy (DR), and diabetic peripheral neuropathy (DPN) were assessed. Predictors were identified using XGBoost and Cox regression, and the impact of lifestyle and multifactorial interventions (MFI) was analyzed.

**Results:**

The prevalence of DKD, DR, and DPN were 39.5%, 26.2%, and 27.1%, respectively, with incidences of 74, 21, and 28 per 1000-person year. XGBoost identified that diabetes duration, age, HbA1c, FBG, triglyceride, BP, serum creatinine, proteinuria, aspirin and statin use were associated with those microvascular complications. The risk of DKD increased more rapidly as HbA1c exceeded 7.5% and decreased as blood pressure was maintained below 120/70 mmHg. Cox regression models showed that community-based intervention, including lifestyle modifications, were associated with a lower risk of DR and DPN. The study also found that higher variability in HbA1c and albumin-to-creatinine ratio (ACR) was associated with an increased risk of microvascular complications.

**Conclusions:**

Community-based interventions significantly reduce the of DR and DPN, highlighting the need for individualized glycemic and BP management in primary care. The findings emphasize the importance of comprehensive management strategies to prevent the development and progression of microvascular complications in type 2 diabetes patients.

**Clinical trial registration:**

http://www.chictr.org.cn/, identifier ChiCTR-TRC-13003222.

## 1 Introduction

The prevalence of diabetes is increasing worldwide, and it is estimated that around 536.6 million adults were living with diabetes in 2021 (1). A significant proportion of diabetic patients complicate with macrovascular complications and microvascular complications. Among these complications, microvascular complications, including diabetic kidney disease (DKD), diabetic retinopathy (DR), and diabetic peripheral neuropathy (DPN) are the most common and debilitating. Their incidences are rarely reported in China. Furthermore, timely diagnosis and treatment are crucial for improving the prognosis of diabetic patients (2, 3). The fundamental task is to identify the modifiable risk factors (4).

Community clinics are major healthcare providers for patients in the world including in China. We reported a cluster-randomized intervention study which found community-based multifactorial intervention improved the overall atherosclerotic cardiovascular disease (ASCVD) risk factors (5). However, the most interesting question was whether these cluster interventions reduced individual diabetic complications. The existing studies have provided a certain insight into the risk factors for microvascular complications, but the results are often conflicting and lack consensus (4, 6). Therefore, there is an urgent need to develop accurate and reliable predictive models to identify the risk factors and prevention strategies for microvascular complications in diabetic patients.

This study was designed to examine the incidence of diabetic microvascular complications, identify the associated risk factors, and evaluate the effectiveness of interventions. We enrolled 3008 patients from 31 community clinics in China and applied both cross-sectional and longitudinal data to identify the factors associated with microvascular complications. The XGBoost model was chosen for the longitudinal cross-sectional analysis to identify the key predictive factors of the occurrence of diabetic microvascular complications (DKD, DR, and DPN) during the 4 years follow-up. This model is particularly suitable for handling complex, non-linear relationships and interactions between variables, making it an excellent choice for identifying important predictors in a large dataset. The Cox regression model was used for the longitudinal analysis to evaluate the risk factors for the development of diabetic microvascular complications over time. This model is well-suited for survival analysis and can handle censored data, allowing us to assess the impact of various factors on the incidence of complications during the follow-up period.

## 2 Materials and methods

### 2.1 Study subjects

Patients with type 2 diabetes who were admitted to 31 community clinics in Beijing and Hebei were enrolled in this study. The inclusion criteria were as follows: diagnosis of type 2 diabetes mellitus was based on the diagnostic criteria of the World Health Organization (WHO) in 1999, and patients who aged 18 - 80 years old. The exclusion criteria were: 1) severe cardiovascular, liver or kidney diseases, 2) end-stage cancers, 3) pregnancy, 4) patients deemed to have poor compliance by the doctors during the initial assessment, including patients who were unlikely to adhere to the study protocols, follow-up schedules, or treatment plans. This analysis is based on the results from 4-years follow-up.

Our study was designed to be a 3-arm community clinics cluster-randomized controlled trial (ClinicalTrial protocol ID ChiCTR-TRC-13003222, http://www.chictr.org.cn/), including control, multiple cardiovascular factors intervention (MFI) and the exercise intervention group. A cluster-randomized design was employed to randomize all the community clinics into the 3 groups by a computer assisted software. The MFI group received comprehensive management for multiple cardiovascular risk factors (including blood glucose, blood pressure, and blood lipids) following the national guideline (5) in addition to traditional lifestyle interventions. The exercise intervention group received an exercise prescription, wearable devices to track physical activity, and guidance based on the collected data, along with traditional lifestyle interventions. However, due to technical limitations with the wearable devices, the exercise intervention group did not receive the planned level of intervention. Consequently, the follow-up frequency for the MFI group and the exercise intervention group became similar, with more frequent follow-ups (every 3 months in the first year, every 6 months in the second year, and every 12 months in the third and fourth years) compared to the control group (every 12 months throughout the 4-year study). Given these circumstances, we combined the MFI group and the exercise intervention group into one intervention group for the analysis. The intervention group was compared to the control group to assess the impact of the interventions on the incidence of microvascular complications.

### 2.2 Data collection

All patients signed an informed consent prior to data collection. A standard questionnaire was administered by doctors in community clinics, including questions related to the personal education, family income, medical insurance status, medical history, and current treatment. Body weight, height, waist circumference, BP were measured. Ankle/brachial index, carotid artery ultrasonography, and 10g nylon filament sensation examination were performed. Blood samples were collected after an overnight (10-14 h) fasting, and the laboratory tests were conducted in the local hospital, including liver function, renal function, fasting blood glucose (FBG), and lipid profiles (total cholesterol (TC), low-density lipoprotein cholesterol (LDL-C), high-density lipoprotein cholesterol (HDL-C), triglyceride (TG)). HbA1c and urine albumin/creatinine ratio (ACR) were determined in central laboratory. Retinopathy status was assessed by fundus photography, and all images were graded by an experienced ophthalmologist. All the laboratories participated in the quality control program as requested by the authority. All data were automatically downloaded from hospital information system. Microvascular complications were evaluated for all patients every year.

### 2.3 Diagnosis of diabetic microvascular complications

DKD was diagnosed based on the presence of albuminuria (ACR ≥ 30mg/g) and/or a reduced estimated glomerular filtration rate (eGFR < 60 mL/min/1.73m^2^) in the absence of signs or symptoms of other kidney diseases (7). Diagnostic criteria of DR were based on the worse eye according to international clinical diabetic retinopathy and diabetic macular edema disease severity scales published in 2002 (8). DPN was diagnosed based on vibration perception threshold and 10 g monofilament test.

### 2.4 Statistical analysis

Standard descriptive statistics (mean and standard deviation for normal continuous variable, median and interquartile range for skewed continuous one, while frequencies and percentages per category for categorical one) were used to demonstrate baseline characteristics of patients.

We conducted a longitudinal cross-sectional analysis using the machine learning algorithm which allowed us to assess the predictive power of various risk factors for microvascular complications over the 4-year follow-up period. By incorporating multiple time points, we aimed to capture the dynamic nature of risk factors and their impact on the development of microvascular complications. We selected three machine learning algorithms, Extreme Gradient Boosting (XGBoost), Logistic Regression (LR), and Support Vector Machine (SVM) to build the models with all the variables as predictors in this study (shown as **Supplementary Table 1**). According to the receiver operating characteristic curves (ROC) and their corresponding area under the curve (AUC) values, XGBoost was employed to develop predictive models for DKD, DR, and DPN finally. The models were evaluated using ten-fold cross-validation to ensure generalizability. This involved randomly dividing the study population into ten groups, where nine groups were used for model training and the remaining one group for testing. This process was repeated ten times, resulting in ten ROCs and AUC values. The average AUC was used to assess the predictive performance of the models, and the confidence interval (CI) of the AUC was used to evaluate the stability of the models. Furthermore, SHAP (Shapley Additive exPlanations) was utilized to interpret the predictive models developed for each microvascular complication (DKD, DR, and DPN). By utilizing SHAP, we were able to gain insights into the specific contributions of different features to the prediction. It helped identify the key features that had the most significant impact on the occurrence of these complications in individuals with type 2 diabetes.

The longitudinal analysis employed a survival analysis paradigm. We mixed the MFI group and exercise intervention group into one group as the intervention group. Cox regression models were employed to develop survival analysis models for DKD, DR, and DPN. The models were evaluated using the average concordance index (C-index) through ten-fold cross-validation. Additionally, recursive feature elimination was applied to select features and simplify the models, thus identifying the significant risk factors associated with each microvascular complication.

The study considered the following variables as potential predictors of diabetic microvascular complications: gender, age at onset of type 2 diabetes mellitus, duration of diabetes, family history, height, weight, systolic blood pressure (SBP), diastolic blood pressure (DBP), blood lipids, blood glucose, HbA1c, liver function, uric acid (UA), medication history, smoking and alcohol history, ACR, eGFR, creatinine, group (definitions of variables are detailed in the **Supplementary Material**). To handle missing data, we conducted a comprehensive analysis of missing data patterns and potential mechanisms. We found that the missing data were primarily missing at random (MAR) and missing completely at random (MCAR). Based on these findings, we chose to use multiple imputation by chained equations (MICE) to impute missing values. This method is appropriate for handling missing data in a wide range of variables and can provide more accurate estimates than simple mean imputation. We performed multiple imputation sensitivity analyses to assess the impact of missing data on key findings. The results of these analyses showed that the key findings were robust and not significantly affected by the missing data.

## 3 Results

### 3.1 Clinical characteristics of patients with type 2 diabetes mellitus

A total of 3,008 patients with type 2 diabetes mellitus were involved in this study. There were 1305 (43.4%) male patients and 1703 (56.6%) female patients. The baseline age, duration of disease, BMI, and HbA1c were 59.7 ± 9.8 years old, 7.6 ± 6.3 years, 25.9 ± 3.3 kg/m^2^, and 7.4 ± 1.6% (57 mmol/mol), respectively. Despite the interventions, the prevalence and incidence of microvascular complications remained high. There were 817 patients who were diagnosed with DKD, 667 with DR, and 663 with DPN at the baseline. A total of 1189 patients with DKD, 788 with DR, and 815 with DPN were diagnosed throughout the follow-up. The prevalence of DKD, DR, and DPN combined baseline and follow-up stages were 39.5%, 26.2%, and 27.1% respectively. And incidences were 74, 21, and 28 per 1000-person year, respectively. In other words, more than one tenth patients develop microvascular complications every year. The clinical characteristics of all the patients were shown in **Table 1**.

**Table 1:** Clinical characteristics (mean value) of T2DM patients according to different diabetic complications (n = 3008, events that occurred throughout the follow-up).

	Total (n=3008)	DKD positive (n=1189)	DKD negative (n=1819)	DR positive (n=788)	DR negative (n=2220)	DPN positive (n=815)	DPN negative (n=2193)
Age (years)	61.1 ± 9.6	61.9 ± 9.6	60.7 ± 10.0^*^	61.1 ± 8.9	61.2 ± 10.2	62.8 ± 8.4	60.5 ± 10.3^*^
Gender [Male, n (%)]	1305 (43.4%)	472 (39.7%)	822 (45.8%)^*^	323 (41.0%)	981 (44.2%)	337 (41.3%)	967 (44.1%)
Duration (years)	8.9 ± 6.3	9.6 ± 6.5	8.5 ± 6.2^*^	10.5 ± 6.4	8.3 ± 6.2^*^	10.3 ± 6.4	8.4 ± 6.2^*^
BMI (kg/m2)	25.9 ± 3.30	26.1 ± 3.4	25.7 ± 3.2^*^	25.8 ± 3.3	25.9 ± 3.3	25.8 ± 3.4	25.9 ± 3.3
Smoking history	27.2%	26.9%	27.4%	26.6%	27.4%	27.2%	27.2%
HbA1c (%)	7.3 ± 1.3	7.6 ± 1.4	7.1 ± 1.3^*^	7.6 ± 1.4	7.2 ± 1.3^*^	7.5 ± 1.3	7.2 ± 1.3^*^
SBP (mmHg)	128.9 ± 9.1	130.4 ± 8.9	128.0 ± 9.1^*^	129.6 ± 9.5	128.7 ± 8.9^*^	131.2 ± 9.6	128.1 ± 8.7^*^
DBP (mmHg)	78.8 ± 6.1	79.0 ± 9.1	78.4 ± 6.3^*^	78.8 ± 6.1	78.8 ± 6.1	79.2 ± 5.8	78.6 ± 6.2^*^
TC (mmol/l)	4.8 ± 1.0	4.9 ± 1.0	4.7 ± 1.0^*^	4.8 ± 0.9	4.8 ± 1.0	4.8 ± 0.9	4.8 ± 1.0
TG (mmol/l)	1.9 ± 1.4	2.1 ± 1.6	1.8 ± 1.3^*^	1.9 ± 1.3	1.9 ± 1.5	1.8 ± 1.2	1.9 ± 1.5
HDL-c (mmol/l)	1.2 ± 0.3	1.2 ± 0.3	1.3 ± 0.3	1.3 ± 0.3	1.2 ± 0.3	1.3 ± 0.3	1.2 ± 0.3
LDL-c (mmol/l)	2.7 ± 0.7	2.8 ± 0.7	2.7 ± 0.7^*^	2.7 ± 0.7	2.7 ± 0.7	2.7 ± 0.7	2.7 ± 0.7
ACR (mg/g)	14.7 (6.4, 34.4)	46.2 (27.2,99)	7.9 (3.5,12.3) ^*^	19.9 (8.7,28.5)	12.3 (5.7,28.5) ^*^	22.7 (10.9,63.7)	11.4 (5.2,26.8) ^*^
eGFR	130.0 ± 33.7	124.0 ± 40.1	134.1 ± 33.8^*^	128.1 ± 31.5	130.7 ± 34.4	126.3 ± 30.2	131.4 ± 34.8^*^

^*^Compared with the former column.

Among the 2131 patients initially free of DKD, 372 patients developed DKD during the follow-up period. The average duration of follow-up was 2.4 years (interquartile range, IQR, 1.3-3.6), and the mean duration of diabetes was 7.4 years (IQR, 2.8-10.6). Similarly, there were 2373 patients initially without DR, of which 121 developed DR during the follow-up period. The average duration of follow-up was 2.6 years (IQR, 1.7-3.7), and the mean duration of diabetes was 7.2 years (IQR, 2.5-10.3). There were 2211 patients initially without DPN, of which 152 developed DPN during the follow-up period. The average duration of follow-up was 2.5 years (IQR, 1.5-3.6), and the mean duration of diabetes was 7.2 years (IQR, 2.6-10.4). Baseline clinical characteristics of those patients who were initially free of diabetic complications were shown in **Table 2**.

**Table 2:** Baseline clinical characteristics of those patients who were initially free of diabetic complications.

	DKD positive n=372	DKD negative n=1760	DKD pvalue	DR positive n=121	DR negative n=2098	DR pvalue	DPN positive n=152	DPN negative n=2060	DPN pvalue
Age (years)	59.7 ± 9.3	59.7 ± 9.8	0.937	58.6 ± 8.1	59.9 ± 10.1	0.162	58.7 ± 7.2	59.4 ± 10.2	0.372
Gender [Male, n (%)]	39.5%	45.4%	0.039	35.5%	44.0%	0.069	40.8%	43.9%	0.455
Duration (years)	7.6 ± 6.2	7.4 ± 6.2	0.581	7.6 ± 6.2]	7.1 ± 6.2	0.436	7.5 ± 5.9	7.2 ± 6.3	0.632
BMI (kg/m2)	26.1 ± 3.3	25.7 ± 3.4	0.092	25.0 ± 4.0	25.9 ± 3.3	0.009	26.2 ± 3.0	25.9 ± 3.4	0.286
Smoking history	26.3%	27.6%	0.614	26.4%	27.8%	0.738	34.9%	27.2%	0.043
HbA1c (%)	7.4 ± 1.6	7.2 ± 1.6	0.043	7.5 ± 1.6	7.3 ± 1.6	0.093	7.5 ± 1.7	7.3 ± 1.6	0.247
SBP (mmHg)	129.8 ± 11.3	127.4 ± 11.7	<0.001	127.7 ± 10.6	128.3 ± 11.7	0.963	130.9 ± 11.6	127.7 ± 11.7	<0.001
DBP (mmHg)	79.7 ± 7.7	78.6 ± 8.1	0.009	77.5 ± 7.5	78.9 ± 8.1	0.210	80.8 ± 7.6	78.9 ± 8.4	0.001
TC (mmol/l)	5.0 ± 1.1	4.9 ± 1.1	0.115	4.8 ± 1.1	5.0 ± 1.2	0.230	5.0 ± 0.9	5.0 ± 1.2	0.776
TG (mmol/l)	1.9 ± 1.5	1.9 ± 1.7	0.492	1.7 ± 1.1	2.0 ± 1.8	0.009	1.9 ± 1.5	2.0 ± 1.9	0.256
HDL-c (mmol/l)	1.3 ± 0.3	1.3 ± 0.3	0.950	1.3 ± 0.3	1.3 ± 0.4	0.857	1.3 ± 0.3	1.2 ± 0.4	0.008
LDL-c (mmol/l)	2.8 ± 0.9	2.8 ± 0.9	0.214	2.7 ± 0.8	2.8 ± 0.9	0.274	2.8 ± 0.8	2.8 ± 0.9	0.580
ACR (mg/l)	11.4 (5.5, 18.0)	7.1 (2.2,13.1)	<0.001	13.7 (6.2, 41.9)	10.2 (4.1, 23.8)	0.004	10.7 (5.7, 22.5)	10.3 (3.6, 25.3)	0.343
eGFR	126.4 ± 37.2	127.6 ± 34.3	0.579	136.1 ± 38.1	125.4 ± 36.4	0.003	127.6± 31.1	126.0 ± 37.4	0.602
HR	64.8 ± 14.4	64.0 ± 15.3	0.596	68.2 ± 10.9	64.1 ± 15.8	0.042	71.5 ± 14.7	62.5 ± 17.0	<0.001
DR	25%	18.7%	0.006	100%	0%	<0.001	32.9%	18.3%	<0.001
DKD	100%	0%	<0.001	30.6%	22.5%	0.040	21.1%	22.9%	0.605
DPN	30.9%	17.7%	<0.001	29.8%	19.1%	0.004	100%	0%	<0.001
2nd relative with T2DM	8.6%	8.3%	0.848	7.4%	8.1%	0.793	12.5%	7.2%	0.017
blurred vision	30.1%	28.3%	0.486	39.7%	27.1%	0.003	30.9%	25.8%	0.169
Nonpalpable dorsalis pedis pulse (L)	5.4%	3.6%	0.118	4.1%	4%	0.945	5.9%	1.3%	<0.001
Nonpalpable dorsalis pedis pulse (R)	5.4%	3.4%	0.070	5.8%	3.5%	0.198	4.6%	1.3%	0.001
edema	9.1%	5.7%	0.013	5%	6.8%	0.427	9.9%	5.5%	0.028
stroke	16.7%	10.2%	<0.001	8.3%	11%	0.345	13.2%	10%	0.216
medication compliance	77.4%	83.2%	0.008	79.3%	83.6%	0.222	63.2%	86.4%	<0.001
fatty liver	24%	29%	0.062	16.5%	28.8%	0.004	30.9	28.3%	0.492
taken statins for over 6m	30.4%	31%	0.818	20.7%	31.5%	0.012	18.4%	33%	<0.001

^*^Compared with the former column.

### 3.2 Longitudinal cross-sectional analysis of predictive factors for microvascular complications in diabetes

**Figure 1:**
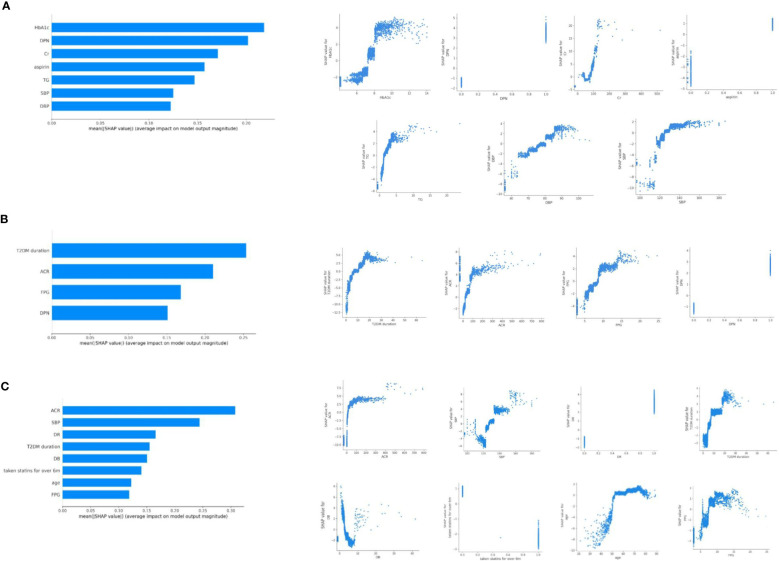
Relationship of different variables with DKD **(A)**, DR **(B)**, and DPN **(C)** based on SHAP analysis of feature importance. SHAP dependence plot of the XGboost model depict that how a single variable affects the prediction. SHAP values for specific features that exceed zero suggest an increased risk of DKD **(A)**, DR **(B)**, and DPN **(C)**.

The average AUC for the cross-sectional analysis model of DKD, DR and DPN were 0.736 (95%CI 0.705, 0.771), 0.707 (95%CI 0.658, 0.756), and 0.751 (95%CI 0.710, 0.792), respectively (**Supplementary Figure 1**). These results indicated that the predictive models have good performance and stability in distinguishing the presence of microvascular complications. Based on SHAP analysis of feature importance, the following associations were observed in the cross-sectional analysis (**Figure 1**). DKD was associated with HbA1c, DPN, creatinine, aspirin use, triglyceride, and BP. DR was associated with disease duration, ACR, FBG, and DPN. DPN was associated with ACR, BP, DR, disease duration, direct bilirubin (DB), statins use, age, and FBG. The relationship between each risk factor and the microvascular complications was determined using SHAP dependency plots. We got very interesting results from the scatter plots (**Figure 1**).

#### 3.2.1 DKD increased rapidly in patients with HbA1c >7.5% and BP>120/70 mmHg

The DKD predictive model categorized HbA1c as a risk factor when it exceeded approximately 7.5%. The risk level of HbA1c changed more rapidly within the range of 7.5-8.5%, while the changes were relatively gradual in other ranges. In addition, the risk of DKD increased rapidly when blood pressure was greater than 120/70 mmHg (maximum slope). TG greater than 2.0 mmol/l was also risk factor of DKD. In the range of 1 to 5mmol/l of TG, the risk degree of DKD was almost linearly positive with TG. Creatinine under 40 μmol/l or exceeding 90 μmol/l indicated possible DKD.

#### 3.2.2 DR and DPN shared more similar risk factors in cross-sectional analysis model

SHAP dependency plots of DR showed that patients with duration longer than 5 years, ACR higher than 30mg/g, FBG higher than 8mmol/l may be prone to complicate with DR. As to DPN, duration longer than 5 years, ACR higher than 30mg/g, FBG higher than 8mmol/l, SBP higher than 130mmHg, age over 50 years may be more associated with DPN. In the range of 0 to 100 mg/g of ACR, the risk degree of DR and DPN increased rapidly with ACR.

### 3.3 Cox regression model showed that lifestyle intervention and MFI intervention significantly reduced the incidence of DR and DPN

The average concordance index for the cox regression model of DKD, DR, and DPN were 0.644 (95%CI 0.634, 0.654), 0.696 (95%CI 0.682, 0.710), and 0.780(95%CI 0.766, 0.794), respectively (**Table 3**). Hazard ratios associated with DKD were: DPN [HR 1.420, 95% CI (1.136, 1.776)], HbA1c [HR 1.122, 95%CI(1.05, 1.197)), UA [HR 1.001,95%CI(1.000, 1.002)], edema [HR 1.551, 95%CI(1.086, 2.215)], stroke [HR 1.648,95%CI(1.249, 2.174)], total cholesterol [HR 1.154, 95%CI(1.052, 1.267)], and ACR [HR 1.032, 95%CI(1.024, 1.041)]. Hazard ratios associated with DR were: BMI [HR 0.931, 95%CI(0.877, 0.987)], DBP [HR 0.975, 95%CI(0.951, 0.999)], HbA1c [HR 1.139, 95%CI(1.020, 1.272)], eGFR [HR 1.010,95%CI(1.000, 1.020)], blurred vision [HR 2.027,95%CI(1.387, 2.963)], fatty liver [HR 0.563,95%CI(0.342, 0.928)], group [HR 0.538,95%CI(0.368, 0.788)]. Hazard ratios associated with DPN were: DR [HR 1.901,95%CI(1.353, 2.671)], family history [HR 1.879,95%CI(1.155, 3.057)]), nonpalpable dorsalis pedis pulse [HR 3.582,95%CI(1.812, 7.081)], medication compliance [HR 0.348,95%(0.248, 0.488)], statins use [HR 0.550,95%CI(0.362, 0.837)], heart rate [HR 1.012,95%CI(1.002, 1.022)], group [HR 0.382,95%CI(0.273, 0.534)]. The analysis showed that the intervention group had a significant impact on the outcomes. Specifically, the intervention group was associated with a lower risk of DR [HR 0.538, 95% CI(0.368,0.788)] and DPN [HR 0.382, 95%CI(0.273, 0.534)] compared to the control group. These findings suggest that the combined interventions were effective in reducing the incidence of DR and DPN.

**Table 3:** Cox regression model and risk predictive factors of DKD, DR, and DPN.

	Risk factors	HR (95% confidential interval)	P value
DKD (concordance index 0.644 (95%CI 0.634, 0.654))	DPN (with vs none)	1.420 (1.136, 1.776)	0.002
	HbA1c (%)	1.122 (1.05, 1.197)	<0.001
	UA (mmol/l)	1.001 (1.000, 1.002)	0.028
	Edema (none vs with)	1.551 (1.086, 2.215)	0.016
	Stroke (none vs with)	1.648 (1.249, 2.174)	<0.001
	TC (mmol/l)	1.154 (1.052, 1.267)	0.002
	ACR (mg/g)	1.032 (1.024, 1.041)	<0.001
DR (concordance index 0.696 (95%CI 0.682, 0.710))	BMI (kg/m2)	0.931 (0.877, 0.987)	0.017
	DBP (mmHg)	0.975 (0.951, 0.999)	0.041
	HbA1c (%)	1.139 (1.020, 1.272)	0.021
	eGFR (mL/min/1.73m2)	1.010 (1.000, 1.020)	0.049
	Blurred vision (with vs none)	2.027 (1.387, 2.963)	<0.001
	Fatty liver (none vs with)	0.563 (0.342, 0.928)	0.024
	Group (intervention vs control)	0.538 (0.368, 0.788)	0.001
DPN (concordance index 0.780 (95%CI 0.766, 0.794))	DR (with vs none)	1.901 (1.353, 2.671)	<0.001
	2^nd^ relative with T2DM (with vs none)	1.879 (1.155, 3.057)	0.011
	Nonpalpable dorsalis pedis pulse (L) (with vs none)	3.582 (1.812, 7.081)	<0.001
	Medication compliance (yes vs no)	0.348 (0.248, 0.488)	<0.001
	Taken statins for over 6m (yes vs no)	0.550 (0.362, 0.837)	0.005
	Heart rate (beats/min)	1.012 (1.002, 1.022)	0.016
	Group (intervention vs control)	0.382 (0.273, 0.534)	<0.001

### 3.4 Discordance of DKD and DR exist

Comparison of incidence rates of DKD, DR, and DPN between control group and intervention group were shown in **Supplementary Figure 2**. When DR and DKD were considered as risk factor for each other, no statistical difference was observed (**Supplementary Figure 3**).

### 3.5 Higher ACR and HbA1c variability were more common in patients with DKD/DR/DPN

We also calculated the standard deviation of ACR and HbA1c for patients throughout the follow-up process, and used T-test to compare the difference between the populations with and without DKD/DR/DPN. The statistical results showed that the standard deviation of ACR or HbA1c for patients with DKD/DR/DPN is significantly higher than that for patients without corresponding complications. This means that the ACR or HbA1c level in patients with DKD/DR/DPN fluctuates more during the follow-up period.

## 4 Discussion

Diabetic microvascular complications can manifest at the time of diagnosis or develop gradually over years, particularly in individuals with type 2 diabetes. Identifying and predicting the occurrence of complications in diabetes is very important, which allows for early intervention and reducing risks of end-stage kidney disease, blindness, amputation, and all-cause mortality. However, current risk prediction models for diabetic microvascular complications based on cross-sectional studies have limitations in accurately predicting future occurrences.

In recent years, artificial intelligence (AI) and machine learning techniques have emerged as valuable tools in analyzing data and establishing models. XGBoost, in particular, has shown excellent specificity and sensitivity in various predictive model studies related to diabetes and its complications, outperforming other models (9–11). In our own study, XGBoost also demonstrated the highest AUC compared to LR and SVM models, and we employed the SHAP method to interpret the results. The XGBoost model provided valuable insights into the predictive power of various risk factors for microvascular complications. By incorporating multiple time points, the model captured the dynamic nature of risk factors and their impact on the development of microvascular complications. However, the model’s predictive accuracy may be limited by the availability and quality of the follow-up data.

The machine learning findings in this study are consistent with existing clinical knowledge regarding the risk factors for diabetic microvascular complications. For example, the XGBoost model identified HbA1c, blood pressure, and serum creatinine as significant predictors of DKD, which aligns with previous studies highlighting the importance of glycemic control and blood pressure management in preventing DKD. Similarly, the model identified disease duration, ACR, and FBG as key risk factors for DR, reinforcing the well-established association between these factors and DR development. The identification of direct bilirubin and nonpalpable dorsalis pedis pulse as significant predictors of DPN adds to the existing body of literature on the pathophysiology of DPN. However, the model also highlighted some novel findings, such as the significant impact of aspirin and statin use on the risk of complications, which have not been extensively documented in previous studies, suggesting that adherence to medication regimens and lipid-lowering therapy may play a role in preventing diabetic complications. These findings suggest that the machine learning approach can complement traditional clinical knowledge and provide new insights into the management of diabetic microvascular complications.

XGBoost analysis model of DKD showed a positive correlation between HbA1c with the occurrence of DKD, indicating that as HbA1c levels increased, the predictive model considered the individual to be at a higher risk of DKD. The risk level of DKD changed more rapidly when HbA1c was in the range of 7.5-8.5%.Therefore, it may be more meaningful to actively lower HbA1c levels just below 7.5% to reduce the incidence of DKD. Similarly, the risk of DKD increased rapidly when BP was greater than 120/70 mmHg, indicating greater clinical significance of controlling BP below 120/70 mmHg. For most nonpregnant adult patients with type 2 diabetes, it is recommended to achieve HbA1c targets of < 7% (12) and BP targets of < 130/80 mmHg (13). Actually, accumulating data suggested that achieving an average HbA1c of ≤ 7.5% would minimize the risk of microvascular complications for people with diabetes mellitus (14). Advocating with patients for an HbA1c target of <7% may avoid therapeutic inertia (14), but it may be unrealistic especially in community setting. The Systolic Blood Pressure Intervention Trial demonstrated that targeting a SBP of less than 120 mmHg decreased cardiovascular event rates and all-cause mortality in high-risk patients (15). ACCORD (Action to Control Cardiovascular Risk in Diabetes) blood pressure trial observed that treatment to a target SBP of <120 mmHg decreased stroke rates by 41% in patients with type 2 diabetes (16). Our data suggested looser glycemic control goals and stricter blood pressure control goals to reduce the occurrence of DKD. Within certain limits, TG level could be as low as possible.

SHAP dependency plots of DR and DPN revealed that duration longer than 5 years, FBG higher than 8mmol/l and ACR higher than 30mg/g are risk factors for the both. SBP higher than 130mmHg and age over 50 years are also related to DPN. Furthermore, patients with DPN would be at a higher risk of DR, while patients with DR are prone to be complicate with DPN. Several cross-sectional studies have found that longer diabetes duration, poorer glycemic and microalbuminuria are strongly associated with DR (17–19). A meta-analysis suggested that the duration of diabetes, age, HbA1c, and DR are associated with significantly increased risks of DPN among diabetic patients (20). Another cross-sectional study concluded that age > 50 years, length of diabetes > 10 years, and FBG >200 mg/dl were the main risk factors for DPN, which was similar to our results (21).

Longitudinal study models have identified multiple predictive factors for DKD, DR, and DPN, most of which are similar to other research findings. Interestingly, the combined interventions, including the MFI and the exercise intervention, were effective in reducing the incidence of DR and DPN. The more frequent follow-ups in the intervention group may have contributed to better management of risk factors and improved adherence to the interventions. However, the technical limitations with the wearable devices in the exercise intervention group should be noted as a potential limitation of the study. Despite these limitations, the findings highlight the importance of comprehensive management strategies in reducing the incidence of microvascular complications in type 2 diabetes patients. Good medication compliance including aspirin and statins use were also related to a reduced occurrence of DPN. In brief, lifestyle intervention and standardized treatment are important to reduce diabetic microvascular complications.

DKD and DR are supposed to share similar pathophysiological mechanisms, and are frequently found simultaneously in patients with diabetes. However, discordance of DKD and DR was also discussed (22). For example, SGLT2 inhibitors have been shown to improve DKD outcomes but do not provide additional benefits for DR. Similarly, GLP-1 agonists have demonstrated varying effects on these two complications. In our study, risk factors for DKD and DR were not consistent in both cross-sectional and longitudinal analyses. Moreover, we did not observe that DKD and DR were risk factors for each other. Our lifestyle interventions also had different outcomes for DKD and DR.

A higher HbA1c variability was associated with a higher risk of microalbuminuria (23), DR (24), and DPN (25) in patients with type 2 diabetes. Our data also confirmed that the fluctuation of HbA1c was associated with occurrence of diabetic microvascular complications. In addition, visit-to-visit variability in albuminuria can independently predict long-term renal function deterioration in patients with type 2 diabetes mellitus (26). In our study, ACR fluctuation was also more common in patients with DKD, DR, and DPN. Clinical strategies targeting HbA1c and ACR variability may provide therapeutic methods to ameliorate diabetic microvascular complications in these patients.

Our study possesses several strengths. Firstly, it includes a large sample size of 3008 patients, providing robust statistical power for the analysis. This enhances the reliability and generalizability of the findings to a broader population of patients with type 2 diabetes. Secondly, we utilized machine learning methods, specifically XGBoost and Cox regression models, to develop accurate and robust predictive models for microvascular complications in diabetes. These models demonstrated good performance and stability in identifying the presence of complications, as indicated by the AUC values obtained. By incorporating machine learning approaches, we enhance the precision and efficiency of risk prediction, offering valuable tools for clinical decision-making and patient management.

However, we must acknowledge several limitations. Firstly, the study sample was limited to patients with type 2 diabetes from community clinics in Beijing and Hebei. Therefore, the generalizability of the findings to other populations or healthcare settings may be restricted. Secondly, the diagnostic criteria of DPN based on vibration perception threshold and 10 g monofilament test were not accurate methods. Thirdly, the accuracy of the predictive models and subsequent analysis may have been affected by missing data or measurement errors. Future research should focus on validating these models in different populations and healthcare settings, while also striving to minimize data limitations and enhance predictive model accuracy. Our study provides valuable insights into the risk factors for microvascular complications in type 2 diabetes patients. However, the analysis would benefit from a more nuanced approach to temporal effects. Future studies should consider implementing methods to account for time-varying confounding, analyze how changes in risk factors over time influence outcomes, and assess the impact of variable follow-up times on results. Additionally, investigating potential temporal interactions between risk factors could provide more detailed insights into the development of microvascular complications.

## 5 Conclusion

In this study, we confirmed the traditional risk factors for microvascular complications diabetes through a maximum 4-year prospective intervention study. The machine learning model found that DKD increased rapidly in patients with HBA1c >7.5% and BP>120/70 mmHg, suggesting a looser glycemic target for control and a stricter blood pressure target for type 2 diabetes management. MFI and exercise intervention in community clinics can significantly reduce the occurrence of DR and DPN, and standardized medication including statins is associated with lower rate of DPN. The fluctuation of ACR and HbA1c is also associated with occurrence of diabetic microvascular complications.

## Data Availability

The original contributions presented in the study are included in the article/**Supplementary Material**. Further inquiries can be directed to the corresponding authors.
