# Supplementary material for: Predict and prevent microvascular complications of type 2 diabetes: a cross-sectional and longitudinal study in Chinese communities
Source: Front Endocrinol (Lausanne). 2025 Mar 31;16:1541663. doi: 10.3389/fendo.2025.1541663 (PMC11994441; doi:10.3389/fendo.2025.1541663)
Supplement: Supplementary file 1 [file DataSheet1.docx]

Variables:

gender (female as 0, male as 1), age at onset (continuous variable, year), duration of diabetes (continuous variable, year), waist circumference (continuous variable, cm), heart rate (continuous variable, beats/min), 1st relative with T2DM/2nd relative with T2DM (none as 0, with family history as 1), height (continuous variable, cm), weight (continuous variable, kg), BMI (continuous variable, kg/m^2^), systolic blood pressure (continuous variable, mmHg), diastolic blood pressure (continuous variable, mmHg), TC (continuous variable, mmol/l), LDL-C (continuous variable, mmol/l), HDL-C (continuous variable, mmol/l), TG (continuous variable, mmol/l), ALT (continuous variable, IU/l), AST (continuous variable, IU/l), GGT (continuous variable, IU/l), ALP (continuous variable, IU/l), Direct Bilirubin (continuous variable, μmol/l), Total Bilirubin (continuous variable, μmol/l), Cr (continuous variable, μmol/l), eGFR (continuous variable, mL/min/1.73m2), uric acid (continuous variable, mmol/l), FPG (continuous variable, mmol/l), HbA1c (continuous variable, %), ACR (continuous variable, mg/g), Internal carotid artery intima-media thickness (L) (continuous variable, mm), Internal carotid artery intima-media thickness (R) (continuous variable, mm), smoking history (none as 0, current smoking as 1, quit smoking as 2), smoking time (continuous variable, year), carotid plaque (none as 0, with carotid plaque as 1), carotid stenosis (none as 0, with carotid stenosis as 1), edema (none as 0, with edema as 1), fatty liver (none as 0, with fatty liver as 1), hypertension (none as 0, with hypertension as 1), hyperuricemia (none as 0, with hyperuricemia as 1), stroke (none as 0, with stroke as 1), numbness (none as 0, with numbness as 1), blurred vision (none as 0, with blurred vision as 1), nonpalpable dorsalis pedis pulse (L) (negative as 0, positive as 1), nonpalpable dorsalis pedis pulse (R) (negative as 0, positive as 1), medication compliance (no as 0, yes as 1), taken statins for over 6m (none as 0, with as 1), group (control as 1, multiple cardiovascular factors intervention and the exercise intervention group as 2)


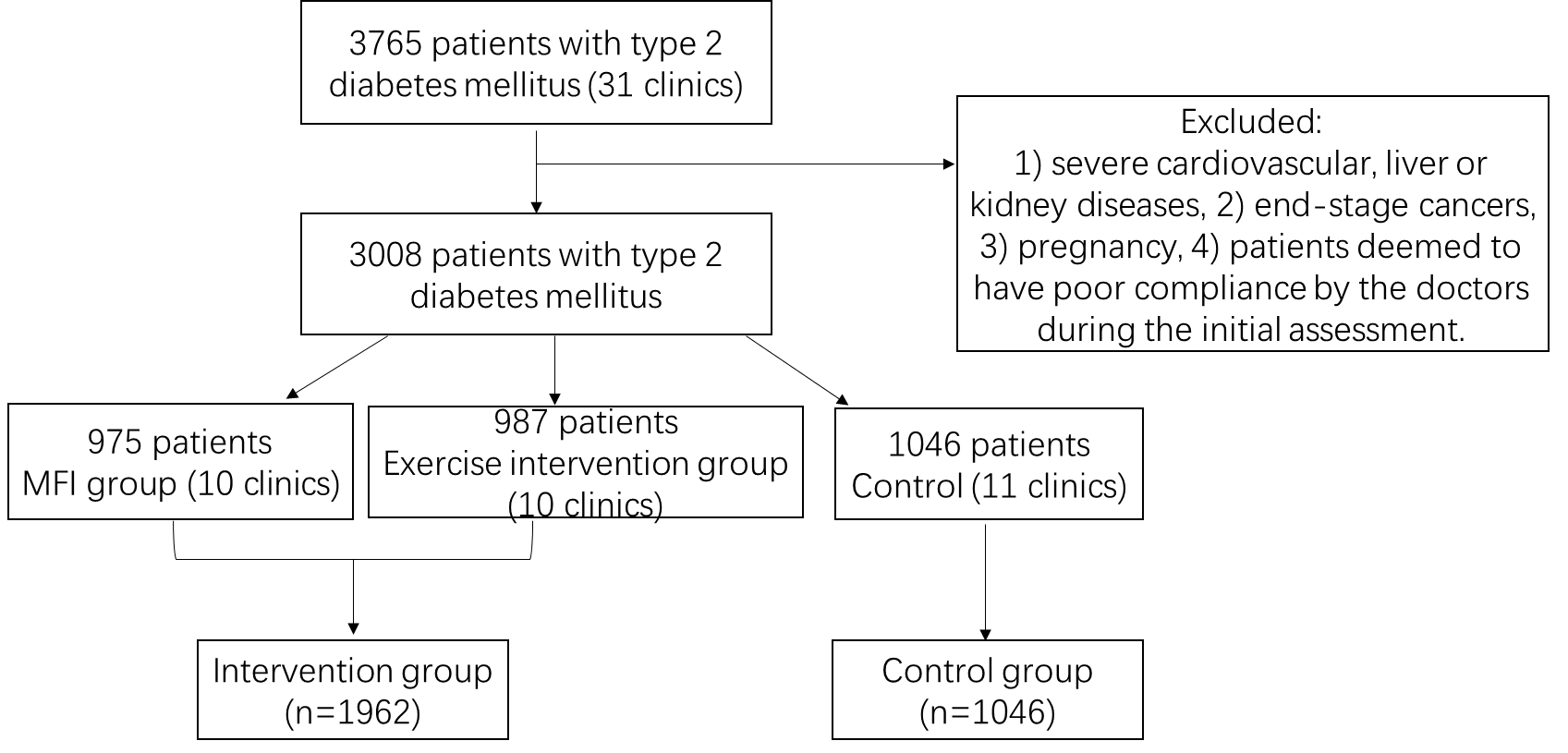


Flowchart of screening patients.

Supplementary table 1 Comparison of mean area under the receiver operating characteristic curves (AUROC) between models based on Extreme Gradient Boosting (XGBoost), Logistic Regression (LR), and Support Vector Machine (SVM).

| Mean AUROC | LR | SVM | XGBoost |
| --- | --- | --- | --- |
| DPN | 0.686 | 0.651 | 0.751 |
| DKD | 0.674 | 0.645 | 0.736 |
| DR | 0.645 | 0.607 | 0.707 |

A B


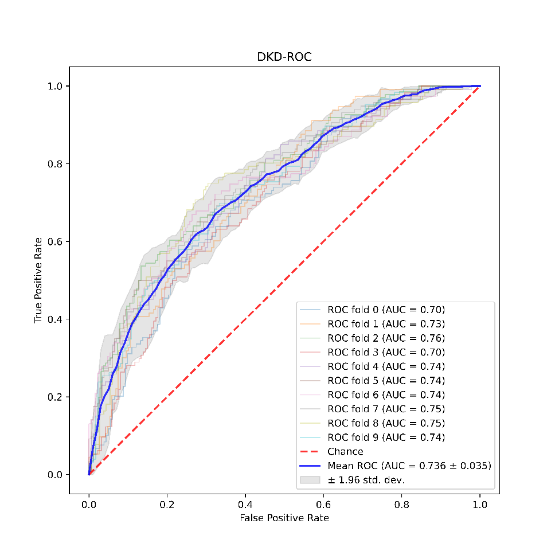

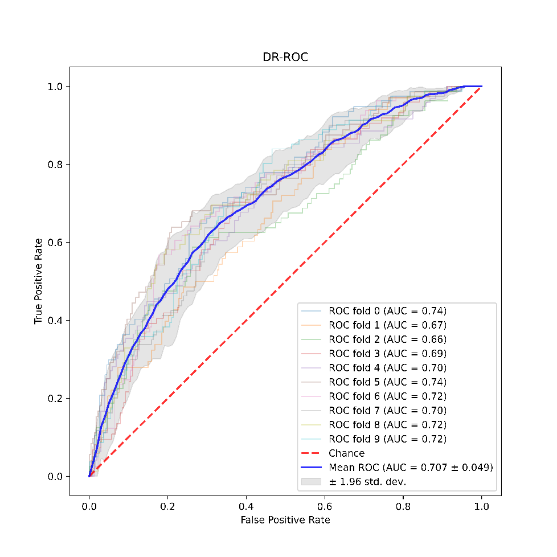


C


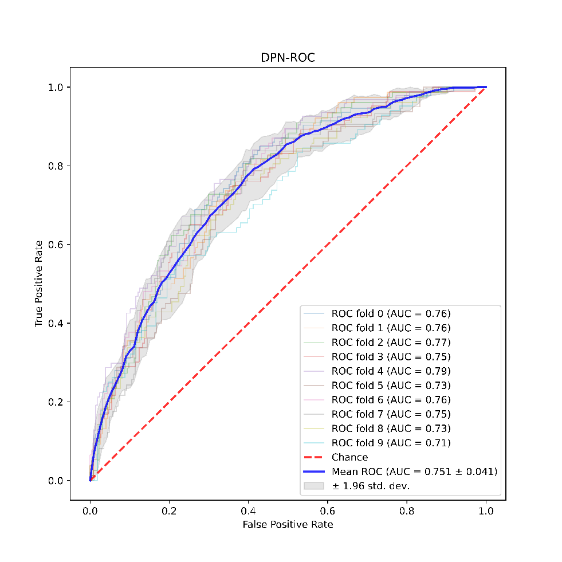


Supplementary Figure 1 Predictive models using XGBoost for DKD (A), DR (B), and DPN (C). The models were evaluated using ten-fold cross-validation to ensure generalizability. The average AUC for the predictive model of DKD, DR and DPN were 0.736 (95%CI 0.705, 0.771), 0.707 (95%CI 0.658, 0.756), and 0.751 (95%CI 0.710, 0.792), respectively.

A (Incidence rate of DKD) B (Incidence rate of DR)


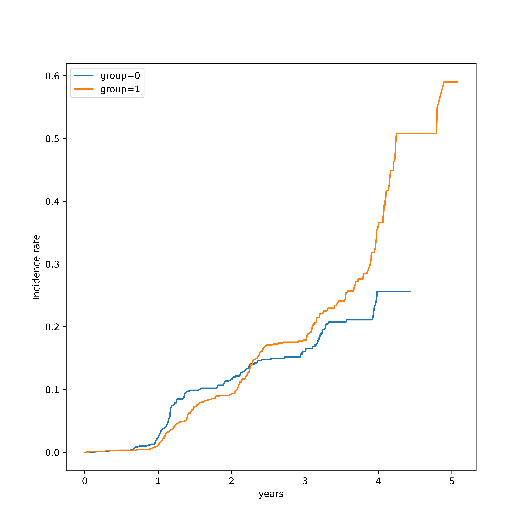

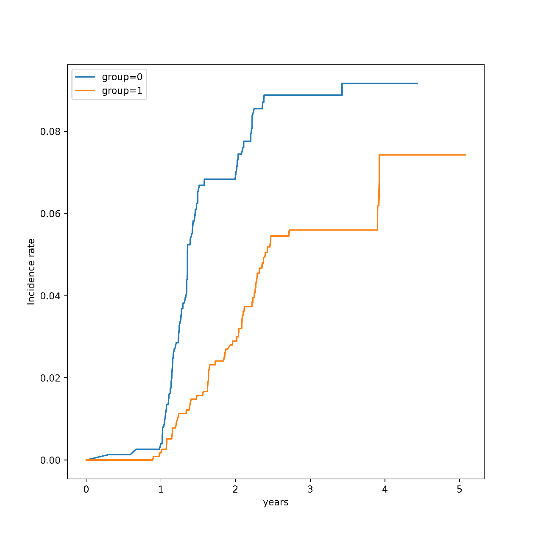


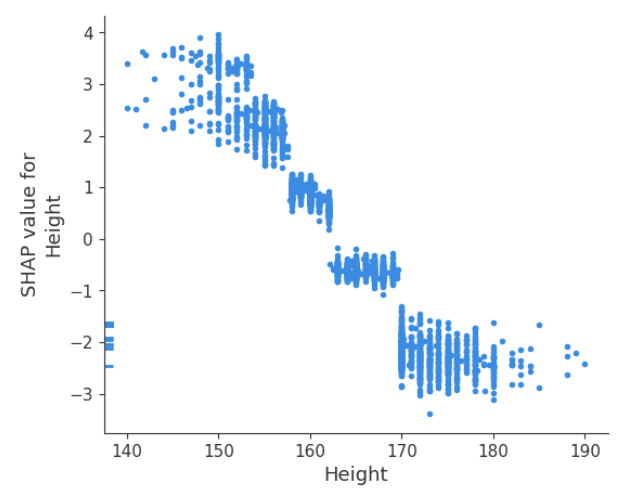

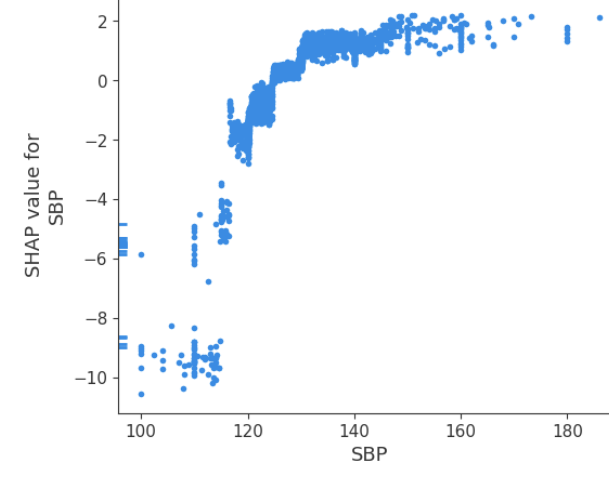
C (Incidence rate of DPN)


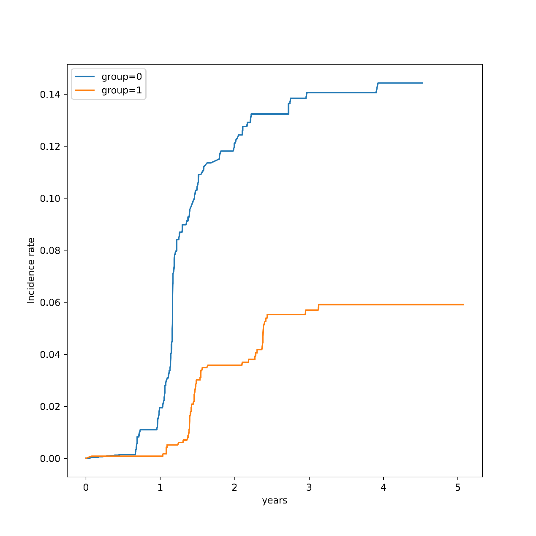


Supplementary Figure 2 Compared with patients in control group (defined as 0), incidence rates of DR (B, p = 0.001) and DPN (C, p < 0.001) decreased significantly in intervention group (defined as 1).


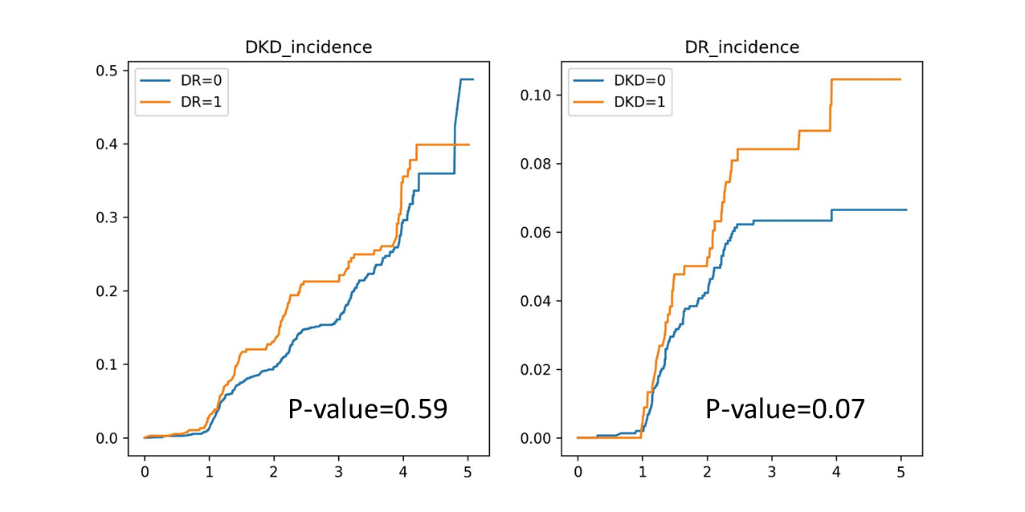


Supplementary Figure 3 DR was not predictive factor for DKD (P=0.59). DKD was not predictive factor for DR (P=0.07).
